# Achieving textbook outcome in liver resection for hepatocellular carcinoma: malnutrition’s pivotal role

**DOI:** 10.1007/s00423-025-03703-x

**Published:** 2025-04-23

**Authors:** Kentaro Oji, Takeshi Urade, Satoshi Omiya, Masahiro Kido, Shohei Komatsu, Hidetoshi Gon, Kenji Fukushima, Hiroaki Yanagimoto, Hirochika Toyama, Takumi Fukumoto

**Affiliations:** Department of Surgery, Division of Hepato-Biliary-Pancreatic Surgery, Kobe University Graduate School of Medicine, Kobe University, 7-5-2 Kusunoki-cho, Chuo-ku, Kobe, 650-0017 Japan

**Keywords:** Carcinoma, Hepatocellular, Liver, Malnutrition, Resection

## Abstract

**Purpose:**

To investigate the impact of textbook outcome (TO) achievement on survival post-liver resection for hepatocellular carcinoma (HCC) and explore the associated factors.

**Methods:**

We retrospectively reviewed 330 patients diagnosed with HCC who underwent initial liver resection at our hospital between January 2011 and December 2019. We also investigated the achievement rates of five TOs and sub-analyzed the relationship between them and malnutrition. The patient’s nutritional status was classified following the Global Leadership Initiative on Malnutrition (GLIM) criteria.

**Results:**

The TO achievement rate was 72.7%. In the prognostic analysis, the TO-achieving group showed significantly longer overall survival (OS) and recurrence-free survival (RFS). Significant differences in age, body mass index, weight loss, muscle mass, serum aspartate aminotransferase level, serum protein induced by vitamin K absence or antagonist-II, tumor characteristics, intraoperative blood loss, perioperative transfusion, and nutritional status were found between the groups.

**Conclusions:**

TO achievement is associated with OS and RFS post-liver resection for HCC. The TO is valuable for evaluating treatment quality in liver resection. Additionally, malnutrition graded following the GLIM criteria, age, tumor stage, and intraoperative blood loss are independent factors for achieving a TO post-liver resection for HCC.

**Supplementary Information:**

The online version contains supplementary material available at 10.1007/s00423-025-03703-x.

## Introduction

Hepatocellular carcinoma (HCC) is the predominant malignant liver tumor and ranks as the third leading cause of cancer death globally, exhibiting a 5-year survival rate of approximately 18% [1, 2]. HCC treatment strategies include chemotherapy and local treatments such as radiofrequency ablation, radiation therapy, and transarterial chemoembolization. However, radical liver resection remains the most effective therapy [3]. Although the morbidity and mortality rates post-liver resection have improved recently with advancements in surgical procedures and perioperative care and management, the overall survival (OS) and recurrence-free survival (RFS) remain unsatisfactory owing to the aging and malnutrition of patients with comorbidities and surgery intolerance [4, 5]. Evaluating treatment quality for older patients or those who are malnourished based solely on a single indicator, such as morbidity or mortality, is insufficient because patients may still experience unfavorable outcomes despite having one indicator improved. Textbook outcome (TO) is a comprehensive evaluation of treatment effects based on multiple surgical outcomes that should be achieved in an ideal postoperative course [6, 7]. TO is suitable for evaluating the impact of treatment in an aging or a malnourished population. While TO is expected to predict the clinical course of several cancers, few studies address TO in HCC, and the analysis of factors involved in achieving TO is also underway. Wu et al. reported that preoperative sarcopenia is associated with TO [8]; however, there is no global consensus on the criteria for sarcopenia, such as the Global Leadership Initiative on Malnutrition (GLIM) criteria (2018), the first reported global standard for nutritional status [9]. The severity of malnutrition, graded according to the GLIM criteria, is reportedly associated with the postoperative prognosis in HCC [10]. Thus, in this study, we aimed to investigate the impact of TO achievement on survival after liver resection for HCC and explore the factors associated with TO achievement. Additionally, the relationship between TO and malnutrition was investigated as the secondary endpoint.

This manuscript is written following the Strengthening the Reporting of Observational Studies in Epidemiology checklist [11].

## Materials and methods

### Participants

Between January 2011 and December 2019, 334 patients with HCC underwent initial liver resection at our hospital. Four patients who did not undergo preoperative plain computed tomography (CT) or had missing weight data were excluded from the study. Finally, 330 patients were included in this study. We retrospectively reviewed the medical records of patients to collect medical data, including age; sex; height; body weight; body mass index (BMI); weight loss; skeletal muscle index at the third lumbar vertebra (L3-SMI); Eastern Cooperative Oncology Group performance status; American Society of Anesthesiologists physical status; Child–Pugh class; indocyanine green retention rate at 15 min (ICGR15); serum aspartate aminotransferase, alanine aminotransferase, alpha-fetoprotein (AFP), and protein induced by vitamin K absence or antagonist II (PIVKA-II) levels; albumin-bilirubin (ALBI) grade; tumor size, number and stage; macroscopic vascular invasion; surgical procedure; surgical approach (minimally invasive or open surgery); operative blood loss; perioperative blood transfusion and complications; and postoperative hospital stay.

The L3-SMIs were calculated to assess muscle mass [12]. The L3-SMI was defined as the cross-sectional area (cm^2^) of the skeletal muscles at the third lumbar vertebral level standardized for height (m^2^). Preoperative liver function was assessed using the Child–Pugh classification and ICGR15. The ALBI score was calculated based on serum albumin and total bilirubin using the following formula: ALBI score = (log10 bilirubin [µmol/L] × 0.66) + (albumin [g/L] × -0.085). Further, the ALBI grade was defined based on the outcome score obtained (≤ -2.60 = Grade 1, -2.60 to ≤ -1.39 = Grade 2, > -1.39 = Grade 3). Tumor stage was diagnosed based on the General Rules for the Clinical and Pathological Study of Primary Liver Cancer [13]. Resection of three or more Couinaud liver segments was defined as major liver resection. Liver resection complications were graded using the Clavien–Dindo classification [14].

### Surgical procedure

Patients with gross ascites, clinical signs of portal hypertension, and extrahepatic metastasis were excluded from liver resection. Both the open and laparoscopic approaches were used for liver resection in this study. Open liver resection was performed via a right subcostal incision with midline extension, while laparoscopic liver resection was routinely performed using five trocars. Laparoscopic liver resection was first performed at our hospital in 2013. The Pringle maneuver was performed intermittently to reduce blood loss using a tourniquet during parenchymal transection. A drainage tube was routinely placed around the cut surface of the remnant liver, followed by wound closure.

### TO

We reviewed previous reports on TO for liver surgery and selected five outcomes as TOs for our study [8, 15–17], including 30-day mortality, surgical margins, 30-day unscheduled readmission rate, major postoperative complications, and prolonged length of postoperative hospital stay (LOS). The tumor margins were classified as microscopically negative (R0). Major postoperative complications were graded using the Clavien–Dindo complication grading system (Grade III or higher), and no prolonged LOS was defined as an LOS of < 20 days (75th percentile of the total cohort). For the LOS threshold, the 75th percentile has been used in several studies on TO [15, 16]; therefore, the same index was used in this study. TO was achieved when the patient satisfied all five outcomes: no 30-day mortality, R0 resection, no 30-day unscheduled readmission, no complication of Clavien–Dindo Grade ≥ III, and no prolonged LOS. Failure to meet at least one criterion for any outcome meant the patient did not achieve TO.

### Assessment of malnutrition

Malnutrition was evaluated using the GLIM criteria [9], including three phenotypic and two etiological criteria. A combination of at least one of both criteria was used to diagnose malnutrition. The etiological criteria contain “cancer.” Patients who met at least one of the phenotypic criteria were diagnosed with malnutrition and classified as having moderate or severe malnutrition. The details of the three phenotypic criteria have been previously reported [10]. The severity of weight loss within 6 months was graded as 5–10% and > 10% for moderate and severe malnutrition, respectively [9]. BMI cutoff values of < 18.5 and < 17.0 kg/m^2^ for patients aged < 70 years and < 20.0 and < 17.8 kg/m^2^ for patients aged 70 years were graded as moderate and severe malnutrition, respectively [18, 19]. Muscle mass reduction was evaluated using L3-SMI from plane CT. L3-SMI cutoff values of < 45.0 and < 37.9 cm^2^/m^2^ in males and < 34.0 and < 28.6 cm^2^/m^2^ in females were evaluated as moderate and severe malnutrition, respectively [20]. Data on weight within 6 months before surgery were collected from self-reported questionnaires, and weight and height were measured during outpatient examinations and inpatient hospitalizations.

### Follow-up

All patients underwent follow-up laboratory tests, including serum AFP and PIVKA-II levels, every 3 months. Additionally, abdominal imaging (contrast-enhanced CT or magnetic resonance imaging) was performed every 3–6 months post-surgery. The patients diagnosed with recurrent HCC were treated following the Clinical Practice Guidelines for the Management of Hepatocellular Carcinoma [21]. All patients were followed up until death, censoring, or December 2023.

### Statistical analyses

All statistical analyses were performed using the JMP software, version 17.0.0 (SAS Institute, Inc., Cary, NC, USA). Continuous variables were expressed as medians, while categorical variables were expressed as absolute numbers (percentages). Differences between groups were evaluated using the Kruskal–Wallis test. Pearson’s chi-square test or Fisher’s exact test was used to analyze categorical variables. In comparing the three cohorts, these tests were corrected using the Bonferroni correction (*p* < 0.0167). OS and RFS were assessed using Kaplan–Meier analysis, and differences between the curves were evaluated using the log-rank test. Univariate and multivariate analyses were performed using Cox proportional hazards regression models to identify variables associated with OS or RFS. Factors with *p* < 0.05 in univariate analysis were included in the multivariate analyses; the tumor stage was the only variable representing the tumor factor. Multivariate analyses were performed using the forced-entry method. Statistical significance was set at *p* < 0.05.

### Ethics

This study was approved by the Institutional Ethics Committee of our Hospital (approval no. B220120) and was conducted following the ethical standards of the Declaration of Helsinki. The requirement for individual consent for this retrospective analysis was waived; an issue explaining the study and an opt-out form are available on the hospital website.

## Results

### Patient characteristics

**Fig. 1:**
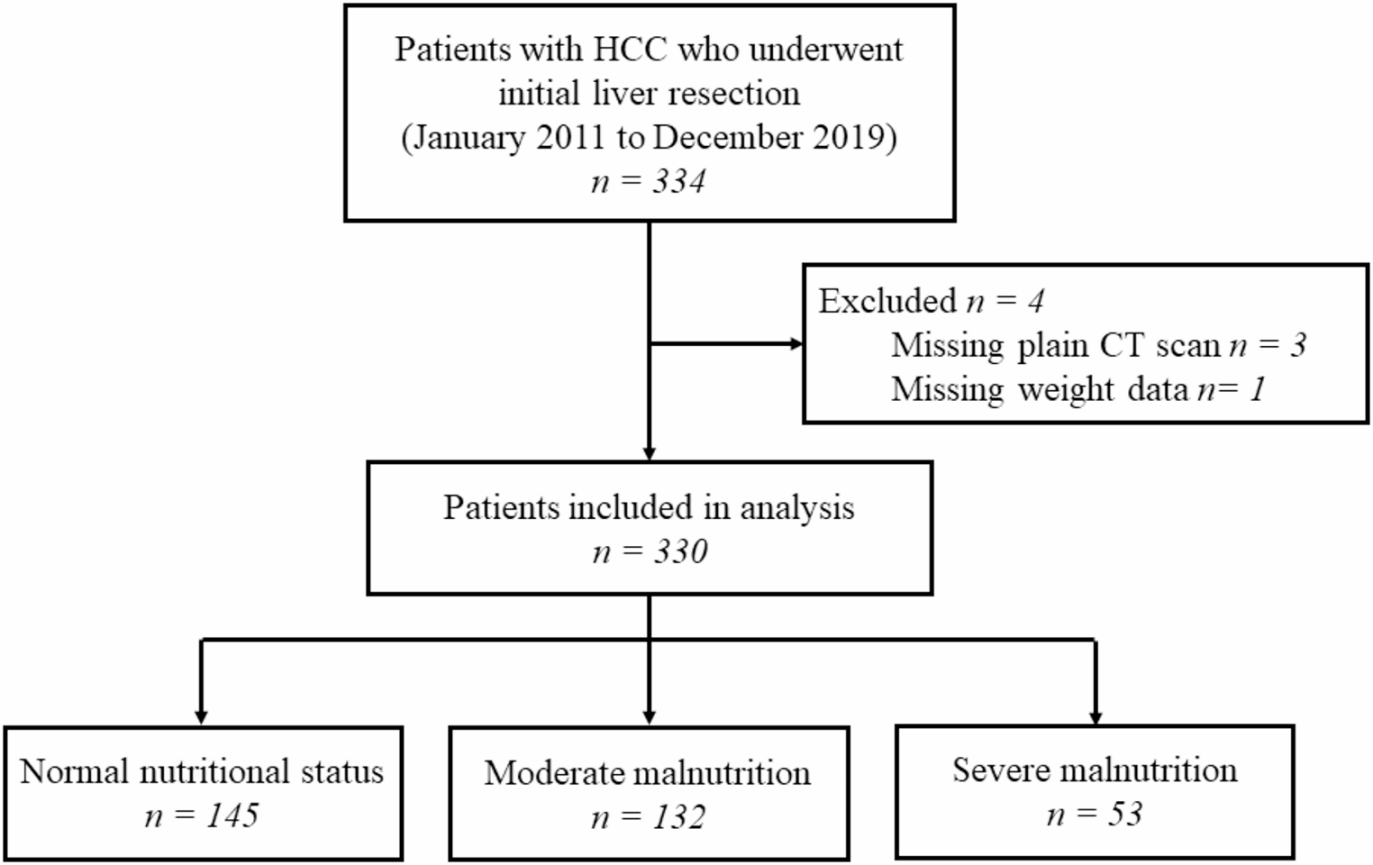
Flowchart for patient selection and grouping according to nutritional status graded using the Global Leadership Initiative on Malnutrition (GLIM) criteria

Table 1 presents the baseline data. Figure 1 shows the patient selection and nutritional status classification using the GLIM criteria as follows: normal nutritional status, 43.9% (*n* = 145); moderate malnutrition, 40.0% (*n* = 132); and severe malnutrition, 16.1% (*n* = 53).

**Table 1:** Clinical characteristics of the entire cohort

Variable		Total *n* = 330 Number (%) or median (range)
Age (years) †		71 (21–93)
Sex	Male	276 (83.6)
	Female	54 (16.3)
Height (m) †		1.64 (1.29–1.82)
Body weight (kg) †		61.6 (34.9–108)
BMI (kg/m^2^) †		22.8 (14.6–39.0)
Weight loss (%) †		1.7 (0–15.8)
L3-SMI (cm2/m2) †		43.2 (25.2–68.9)
ECOG-PS (0/ ≥ 1)	0	307 (93.0)
	≥ 1	23 (7.0)
ASA-PS	≤II	285 (86.4)
	≥III	45 (13.6)
Child-Pugh class	A	320 (97.0)
	B	10 (3.0)
ICGR15 (%) †		11.7 (1.1–61.3)
ALBI grade	≤ 2a	59 (17.9)
	≥ 2b	271 (82.1)
Serum AST (IU/L) †		33 (12–448)
Serum ALT (IU/L) †		29.5 (6–214)
Serum AFP (ng/mL) †		9 (0.8–655,600)
Serum PIVKA-II (mAU/mL) †		220 (12–379,745)
Tumor size (mm) †		40 (10–230)
Tumor number	Solitary	233 (70.6)
	Multiple	97 (29.4)
Macroscopic vascular invasion		55 (16.7)
Tumor stage	≤II	213 (64.5)
	≥III	117 (35.5)
Surgical procedure	Minor	248 (75.2)
	Major	82 (24.8)
Surgical approach	MIS	121 (36.7)
	Open	209 (63.3)
Operative blood loss (mL) †		272.5 (5–4,300)
Perioperative blood transfusion		62 (18.8)
Postoperative hospital stays (days) †		14 (5-130)
Nutritional status graded by the GLIM criteria	Normal	145 (43.9)
	Moderate	132 (40.0)
	Severe	53 (16.1)

Values in parentheses are percentages unless indicated otherwise; †values are median (range)

AFP, alpha-fetoprotein; ALBI, albumin-bilirubin; ALT, alanine aminotransferase; ASA-PS, American Society of Anesthesiologists physical status; AST, aspartate aminotransferase; BMI, body mass index; ECOG-PS, Eastern Cooperative Oncology Group performance status; GLIM; Global Leadership Initiative on Malnutrition; ICGR15, indocyanine green retention rate at 15 min; L3-SMI, skeletal muscle index at the third lumbar vertebra; MIS, minimally invasive surgery; PIVKA-II, protein induced by vitamin K absence or antagonist II

### TO

**Fig. 2:**
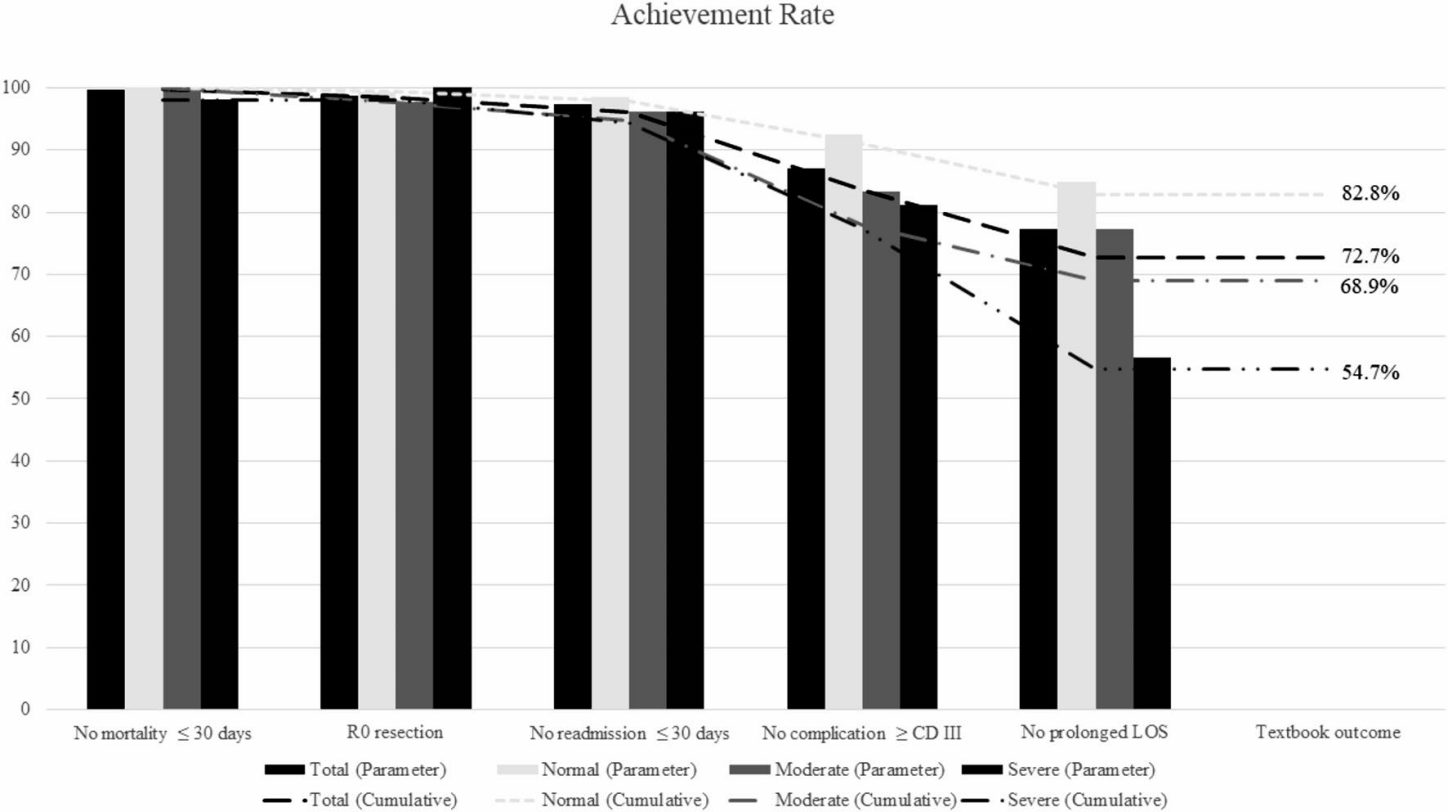
Textbook outcome and individual items distribution

Table 2 compares the characteristics of the TO-achieving group (TO group) with those of the group not achieving TO (non-TO group). Significant differences were observed in age, BMI, weight loss, L3-SMI, ALBI grade, serum AST, serum PIVKA-II, tumor size, number, stage, macroscopic vascular invasion, minimally invasive surgery, operative blood loss, perioperative blood transfusion, postoperative hospital stay, and nutritional status according to the GLIM criteria between the groups. Table 3; Fig. 2 show the five outcomes and achievement rates of TO.

**Table 2:** Comparison of clinical characteristics between TO and non-TO groups

Variable		TO group *n* = 240 Number (%) or median (range)	Non-TO group *n* = 90 Number (%) or median (range)	*P* value
Age (years) †		69 (21–93)	72 (39–91)	0.003
Sex	Male	200 (83.3)	76 (84.4)	0.868
	Female	40 (16.7)	14 (15.6)	
Height (m) †		1.64 (1.29–1.82)	1.64 (1.42–1.79)	0.960
Body weight (kg) †		62.6 (34.9–108)	59.2 (38.8–93.4)	0.059
BMI (kg/m^2^) †		23.1 (15.7–39.0)	21.9 (14.6–33.1)	0.043
Weight loss (%) †		1.55 (0–14.84)	2.11 (0–15.76)	0.009
L3-SMI (cm^2^/m^2^) †		44.2 (25.3–68.9)	40.6 (28.5–57.5)	0.002
ECOG-PS (0/ ≥ 1)	0	224 (93.3)	83 (92.2)	0.808
	≥ 1	16 (6.7)	7 (7.8)	
ASA-PS	≤II	211 (88.0)	74 (82.2)	0.207
	≥III	29 (12.0)	16 (17.8)	
Child-Pugh class	A	235 (97.9)	85 (94.4)	0.143
	B	5 (2.1)	5 (5.6)	
ICGR15 (%) †		11.7 (1.1–61.3)	11.4 (1.7–56.7)	0.915
ALBI grade	≤ 2a	204 (85.0)	67 (74.4)	0.035
	≥ 2b	36 (15.0)	23 (25.6)	
Serum AST (IU/L) †		32 (13–194)	36.5 (12–448)	0.036
Serum ALT (IU/L) †		29 (6–214)	33 (7–149)	0.371
Serum AFP (ng/mL) †		8 (0.8–191,100)	13.5 (1–655,600)	0.273
Serum PIVKA-II (mAU/mL) †		167.5 (12–265,961)	532.5 (16–379,745)	0.005
Tumor size (mm) †		35 (10–230)	48 (12–160)	< 0.001
Tumor number	Solitary	186 (77.5)	47 (52.2)	< 0.001
	Multiple	54 (22.5)	43 (47.8)	
Macroscopic vascular invasion		33 (13.8)	22 (24.4)	0.030
Tumor stage	≤II	174 (72.5)	39 (43.3)	< 0.001
	≥III	66 (27.5)	51 (56.7)	
Surgical procedure	Minor	184 (76.7)	64 (71.1)	0.318
	Major	56 (23.3)	26 (28.9)	
Surgical approach	MIS	99 (41.3)	22 (24.4)	0.004
	Open	141 (58.7)	68 (75.6)	
Operative blood loss (mL) †		210 (5–3,480)	520 (5–4,300)	< 0.001
Perioperative blood transfusion		32 (13.3)	30 (33.3)	< 0.001
Postoperative hospital stays (days) †		13 (5–20)	29 (7–130)	< 0.001
Nutritional status graded by the GLIM criteria	Normal	120 (50.0)	25 (27.8)	< 0.001
	Moderate	91 (37.9)	41 (45.6)	
	Severe	29 (12.1)	24 (26.7)	

Values in parentheses are percentages unless indicated otherwise; †values are median (range)

AFP, alpha-fetoprotein; ALBI, albumin-bilirubin; ALT, alanine aminotransferase; ASA-PS, American Society of Anesthesiologists physical status; AST, aspartate aminotransferase; BMI, body mass index; ECOG-PS, Eastern Cooperative Oncology Group performance status; GLIM; Global Leadership Initiative on Malnutrition; ICGR15, indocyanine green retention rate at 15 min; L3-SMI, skeletal muscle index at the third lumbar vertebra; MIS, minimally invasive surgery; PIVKA-II, protein induced by vitamin K absence or antagonist II; TO, textbook outcome

**Table 3:** Achievement rate of textbook outcome in entire cohort and the comparison between nutritional status cohorts

Outcome	Total *n* = 330 Number (%)	Normal *n* = 145 Number (%)	Moderate *n* = 132 Number (%)	Severe *n* = 53 Number (%)	*P* value
Textbook outcome	240 (72.7)	120 (82.8)	91 (68.9)	29 (54.7)	< 0.001
No mortality ≤ 30 days	329 (99.7)	145 (100)	132 (100)	52 (98.1)	0.072
R0 resection	326 (98.8)	144 (99.3)	129 (97.7)	53 (100)	0.329
No readmission ≤ 30 days	321 (97.3)	143 (98.6)	127 (96.2)	51 (96.2)	0.412
No complication of CD ≥ III	287 (87.0)	134 (92.4)	110 (83.3)	43 (81.1)	0.031
No prolonged LOS	255 (77.3)	123 (84.8)	102 (77.3)	30 (56.6)	< 0.001

CD, Clavien–Dindo classification; LOS, length of postoperative hospital stay

### Survival analysis

**Fig. 3:**
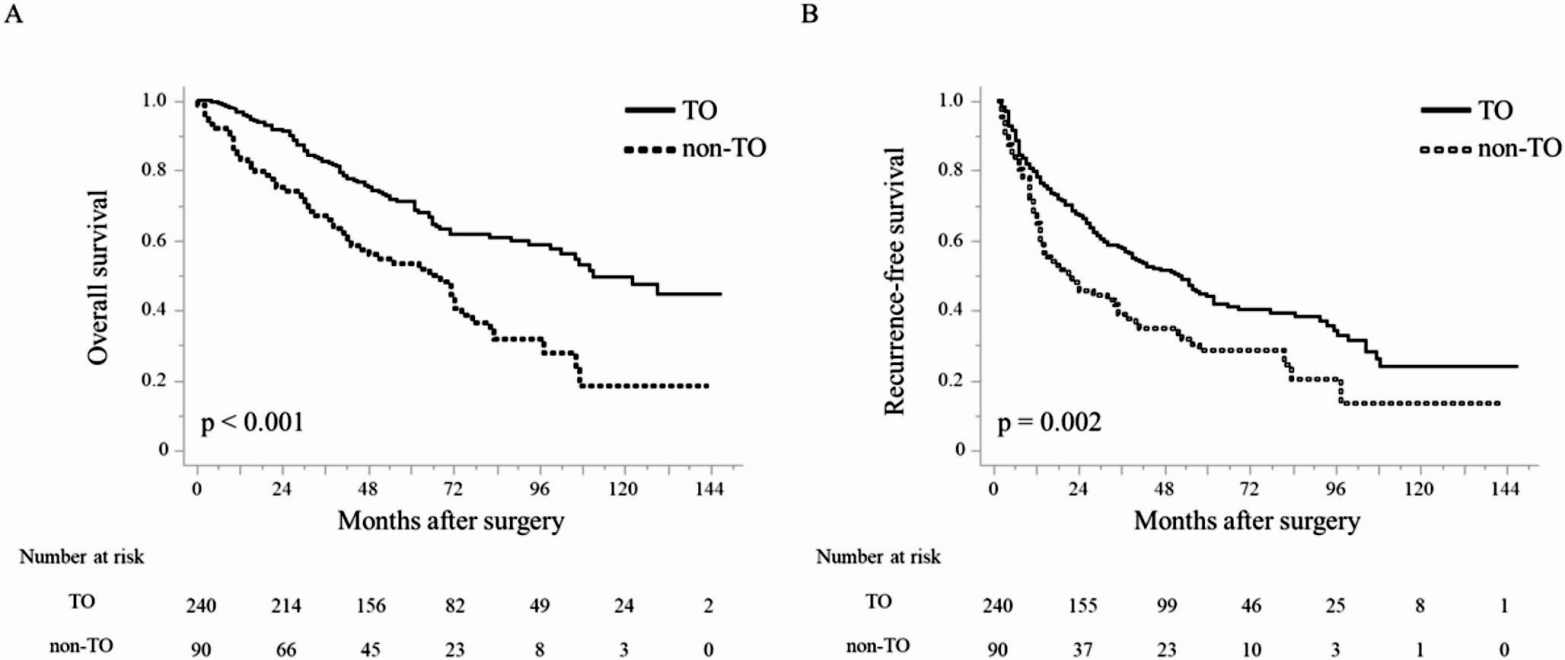
Comparison of patient outcomes according to achievement of textbook outcome. (**a**) Overall survival in the entire cohort (**b**) recurrence-free survival in the entire cohort

The results of the survival analysis shown in Fig. 3 revealed that in the total cohort, OS and RFS were significantly improved in the TO group than in the non-TO group (OS: 111 vs. 68 months, *p* < 0.001; RFS: 52 vs. 20 months, *p* = 0.002, respectively).

### TO-associated factors

To further investigate the factors affecting TO, we used logistic regression analysis (Table 4). Univariate logistic regression analysis showed that age, ALBI grade, serum AST, serum PIVKA-II, tumor number, size, stage, macroscopic vascular invasion, surgical approach, intraoperative blood loss, perioperative blood transfusion, and nutritional status graded according to the GLIM criteria were significantly associated with TO achievement. Multivariate logistic regression analysis showed that age, tumor stage, operative blood loss of > 500 mL, and nutritional status graded according to the GLIM criteria were independent risk factors for TO.

**Table 4:** Univariate and multivariate analysis for textbook outcome achievements

Variables		Univariate OR	95% CI	*P* value	Multivariate OR	95% CI	*P* value
Age (years)	< 70	Reference			Reference		
	≥ 70	0.43	0.26–0.72	0.001	0.37	0.20–0.67	0.001
Sex	Male	Reference					
	Female	1.08	0.55–2.10	0.808			
ECOG-PS	0	Reference					
	≥ 1	0.84	0.33–2.13	0.724			
ASA-PS	≤II	Reference					
	≥III	0.63	0.32–1.23	0.182			
Child-Pugh class	A	Reference					
	B	0.36	0.10–1.28	0.114			
ICGR15 (%)	< 10	Reference					
	≥ 10	1.00	0.54–1.49	0.685			
ALBI grade	≤ 2a	Reference			Reference		
	≥ 2b	0.51	0.28 − 0.92	0.027	0.93	0.46 − 1.89	0.849
Serum AST (IU/L)	≤ 50	Reference			Reference		
	> 50	0.53	0.31–0.92	0.025	0.90	0.47–1.70	0.747
Serum ALT (IU/L)	≤ 50	Reference					
	> 50	0.58	0.32–1.04	0.069			
Serum AFP (ng/mL)	< 20	Reference					
	≥ 20	0.73	0.44–1.19	0.211			
Serum PIVKA-II (mAU/mL)	< 40	Reference			Reference		
	≥ 40	0.41	0.21–0.77	0.006	0.60	0.30–1.22	0.164
Tumor size (mm)	< 50	Reference					
	≥ 50	0.49	0.29–0.80	0.004			
Number of tumors	Solitary	Reference					
	Multiple	0.31	0.19–0.52	< 0.001			
Macroscopic vascular invasion	Negative	Reference					
	Positive	0.49	0.26–0.90	0.021			
Tumor stage	≤II	Reference			Reference		
	≥III	0.29	0.17–0.48	< 0.001	0.39	0.21–0.71	0.002
Surgical procedure (major/minor)	Minor	Reference					
	Major	0.74	0.43–1.29	0.299			
Surgical approach (MIS/open)	MIS	Reference			Reference		
	Open	0.46	0.26–0.79	0.005	1.38	0.67–2.82	0.379
Operative blood loss (mL)	< 500	Reference			Reference		
	≥ 500	0.29	0.17–0.48	< 0.001	0.33	0.16–0.69	0.003
Perioperative blood transfusion	No	Reference			Reference		
	Yes	0.30	0.17–0.54	< 0.001	0.66	0.32–1.38	0.273
Nutritional status graded by the GLIM criteria	Normal	Reference			Reference		
	Moderate	0.46	0.26–0.81	0.007	0.59	0.31–1.11	0.107
	Severe	0.25	0.13–0.50	< 0.001	0.33	0.15–0.72	0.005

AFP, alpha-fetoprotein; ALBI albumin-bilirubin; ALT, alanine aminotransferase; ASA-PS, American Society of Anesthesiologists physical status; AST, aspartate aminotransferase; CI, confidence interval; ECOG-PS, Eastern Cooperative Oncology Group performance status; GLIM; Global Leadership Initiative on Malnutrition; ICGR15, indocyanine green retention rate at 15 min; L3-SMI, skeletal muscle index at the third lumbar vertebra; MIS, minimally invasive surgery; OR, odds ratio; PIVKA-II, protein induced by vitamin K absence or antagonist II

### Nutritional status

In subgroup analyses, the association between nutritional status and TO achievement was examined (Table 3; Fig. 2). Among the groups classified according to the GLIM criteria, there were differences in the total TO, LOS, and major postoperative complications. The median LOS (days) for each group were normal, 13 days; moderate malnutrition, 15 days; and severe malnutrition, 17 days. Significant differences in LOS were observed between the groups (*p* = 0.005). The proportions of Clavien–Dindo Grade ≥ III complications for each nutrition group were normal, 7% (*n* = 11); moderate malnutrition, 17% (*n* = 22); and severe malnutrition, 19% (*n* = 10). OS and RFS of the nutritional subgroups were also analyzed (Supplementary Fig. 1–3). The results showed that OS improved in the TO group compared to the non-TO group in the normal and moderate malnutrition groups (*p* = 0.006 and 0.005, respectively). However, no significant difference in the severe malnutrition group was observed. RFS improved in the TO group compared to the non-TO group, but only in the moderate malnutrition group (*p* = 0.003).

## Discussion

The key findings of the study were as follows. The total rate of TO achievement in our study was 72.7%; in the survival analyses, TO achievement was significantly associated with OS and RFS. Malnutrition graded as per the GLIM criteria was associated with TO achievement and was an independent factor affecting the TO in this study. Age, tumor stage, and intraoperative blood loss were also independent factors for achieving TO post-liver resection for HCC.

The strengths of this study include the uniformity of treatment process, as it was conducted at a single institution, the long-term prognostic follow-up, and the inclusion of records such as preoperative weight changes. However, this study has some limitations. First, this was a small, retrospective, single-center study. A larger sample size, such as in a multicenter study, may more reliably confirm the present results or reveal additional facts. Second, to evaluate nutritional status, the measurement of muscle mass requires manual measurement of the SMI values. New techniques, such as artificial intelligence imaging, are expected to produce uniform results, even among different researchers. Finally, the TO items were not standardized in each liver resection study. While a uniform definition of TO would facilitate comparing treatment effectiveness, the variability of TO items based on research purposes necessitates individual consideration of each item.

Surgical studies usually evaluate a single postoperative indicator (morbidity or mortality). However, poor patient satisfaction often correlates with poor performance of these indicators. TO, proposed by colorectal surgeons from the Netherlands in 2013, is a clear all-or-none indicator and comprehensive evaluation using multiple outcomes, making it suitable for “patient-centered” quality evaluation [7]. This comprehensive evaluation system is more suitable for comparing the overall quality of medical care at an institution than an evaluation using only a single indicator, which may be influenced by regional differences. TO should provide information to patients to make better decisions. In particular, liver resection requires a high level of medical care and is predominantly implemented in high-volume centers [22]. In these centers, as technically feasible and minimally invasive liver resections increase owing to advances in surgical devices and perioperative care, the target patient population is likely to include an increasing number of older and/or malnourished patients with comorbidities [4]. TO has become the most patient-oriented index of liver resection in recent years.

In this study, we defined TO components by referring to previous TO studies on liver resection. LOS is a valid indicator for evaluating the quality of treatment and patient satisfaction; however, the outcome is affected by the insurance system or regional culture. For example, the economic incentives to minimize the LOS are relatively less in Japan compared with that in other countries, because of the comprehensive coverage provided by the National Health Insurance system. Additionally, while rehabilitation and post-discharge care for older adults or unaccompanied patients are often managed in outpatient or transfer facilities in other countries, these services are more commonly provided during hospitalization in Japan, potentially contributing to a prolonged LOS. In a multicenter or international study, it is necessary to consider using a more stringent threshold, such as the 50th percentile, or excluding LOS from the TO component. Whether LOS should be included in TO should be considered depending on the purpose of the study. Complications were defined as Grade III or higher according to the Clavien–Dindo classification in almost all surgical studies. Patients who experienced only complications up to Grade II were considered the TO group in the present study, but we thought they were treated with prolonged drug administration, such as antibiotics, and were reflected in a longer hospital stay. If the LOS was not prolonged, it was considered acceptable as a desirable course. As this study focused on liver resection for HCC, only positive or negative surgical margins were evaluated as oncological indicators. Although the incidences of postoperative death and readmission were low, they were included in TO because they significantly affected the patient’s benefits.

The total rate of TO achievement in the present study was 72.7%, which is similar to that in previous studies (62.0–80.5%) [15–17]. LOS had the most negative impact on TO achievement, followed by complications. Many studies have used these measures as comparators of treatment, indicating that even these single measures have the power to evaluate treatment. However, patients who achieved TO received high-quality treatment with a high degree of satisfaction without any undesirable results. In the survival analyses, TO achievement was significantly associated with OS and RFS. TO impacted not only on short-term results but also on long-term prognosis in liver resection for HCC. It is well known that the complication rate post-liver resection is related to survival post-liver resection for HCC [23], and it is reasonable that TO, as a collection of short-term outcomes, is associated with long-term prognosis.

Nutritional status is an important issue in the increasing number of surgeries performed on older and malnourished patients. Additionally, low skeletal muscle mass correlates with poor clinical outcomes such as postoperative complications, chemotherapy toxicity, and mortality in older patients with cancer [24]. While muscle mass reduction was reported to be associated with the achievement of TO [8], the GLIM criteria are more accurate in diagnosing malnutrition by assessing phenotypes, including weight trends, prior to surgery. Malnutrition graded according to the GLIM criteria was associated with TO achievement as an independent factor in this study. Previous studies have reported that malnutrition is associated with individual outcomes such as complications and prolonged hospital stays, and the results of the present study were in line with theirs [25, 26]. Several studies reported that nutritional status affects not only short-term surgical outcomes but also postoperative survival post-liver resection [10, 27]. In the subgroup analyses of nutritional status, TO achievement was associated with OS in patients with normal nutritional status and moderate malnutrition. In contrast, patients with severe malnutrition had a poorer prognosis regardless of TO achievement and were found to be more difficult to treat, including factors other than surgery and the disease itself [28]. Tumor-associated weight loss and muscle mass reduction may be due to metabolic disturbances caused by inflammation, reflecting the rapid progression of the tumor and the relatively high burden of cancer in the body. The recurrence rate may also be affected by tumor activity, causing malnutrition [29, 30]. Patients with severe malnutrition experience prolonged recovery time, with or without complications. In the severe malnutrition group, the proportion of patients categorized into the non-TO group because of prolonged LOS without complications was higher than that in the other groups (normal, 9.0%; moderate, 9.8%; and severe, 24.5%). Although complications are associated with prognosis, the association between LOS and survival remains unclear. This may explain why there was no survival difference between TO and non-TO patients in the severe malnutrition group. However, TO is essentially a short-term indicator of patient satisfaction, and its impact on prognosis may only be partially considered in severe malnutrition cohorts.

Nutritional interventions such as prehabilitation and preoperative nutritional therapy may improve TO achievement rates and prognosis. However, chronic inflammation and protein catabolism owing to the presence of tumors can lead to nutritional decline [31], and the extent to which preoperative improvement can be expected in the tumor-bearing state remains unclear. Additionally, a prospective study with a uniform protocol is required to evaluate the usefulness of these preoperative interventions.

Postoperative evaluation using TO has proven valuable and is associated with long-term prognosis. Using TO across various treatments is advantageous for evaluating patient-centered treatment in an era of increasing surgeries for patients with aging and various co-morbidities. This study demonstrated a correlation between the amount of blood loss and the rate of TO achievement, indicating that it is crucial to manage intraoperative blood loss. Additionally, nutritional status should be taken into consideration when determining a patient’s indication for liver resection, as malnutrition negatively affects TO. We posit that improving malnutrition through preoperative intervention is effective.

## Conclusions

TO achievement is a prognostic indicator of survival post-liver resection for HCC. Nutritional status graded using the GLIM criteria was associated with TO achievement and was an independent factor for TO achievement post-liver resection for HCC, along with age, tumor stage, and blood loss.

## Electronic supplementary material

Below is the link to the electronic supplementary material.

Supplementary Material 1: Supplementary Fig. 1: Comparison of patient outcomes according to achievement of textbook outcome in the normal nutritional cohort. (a) Overall survival; (b) recurrence-free survival

Supplementary Material 2: Supplementary Fig. 2: Comparison of patient outcomes according to achievement of textbook outcome in the moderate malnutritional cohort. (a) Overall survival; (b) recurrence-free survival

Supplementary Material 3: Supplementary Fig. 3: Comparison of patient outcomes according to achievement of textbook outcome in the severe malnutritional cohort. (a) Overall survival; (b) recurrence-free survival

Supplementary Material 4

## Data Availability

No datasets were generated or analysed during the current study.
