# Supplementary figures and images for: Achieving textbook outcome in liver resection for hepatocellular carcinoma: malnutrition’s pivotal role
Source: Langenbecks Arch Surg. 2025 Apr 23;410(1):139. doi: 10.1007/s00423-025-03703-x (PMC12018603; doi:10.1007/s00423-025-03703-x)

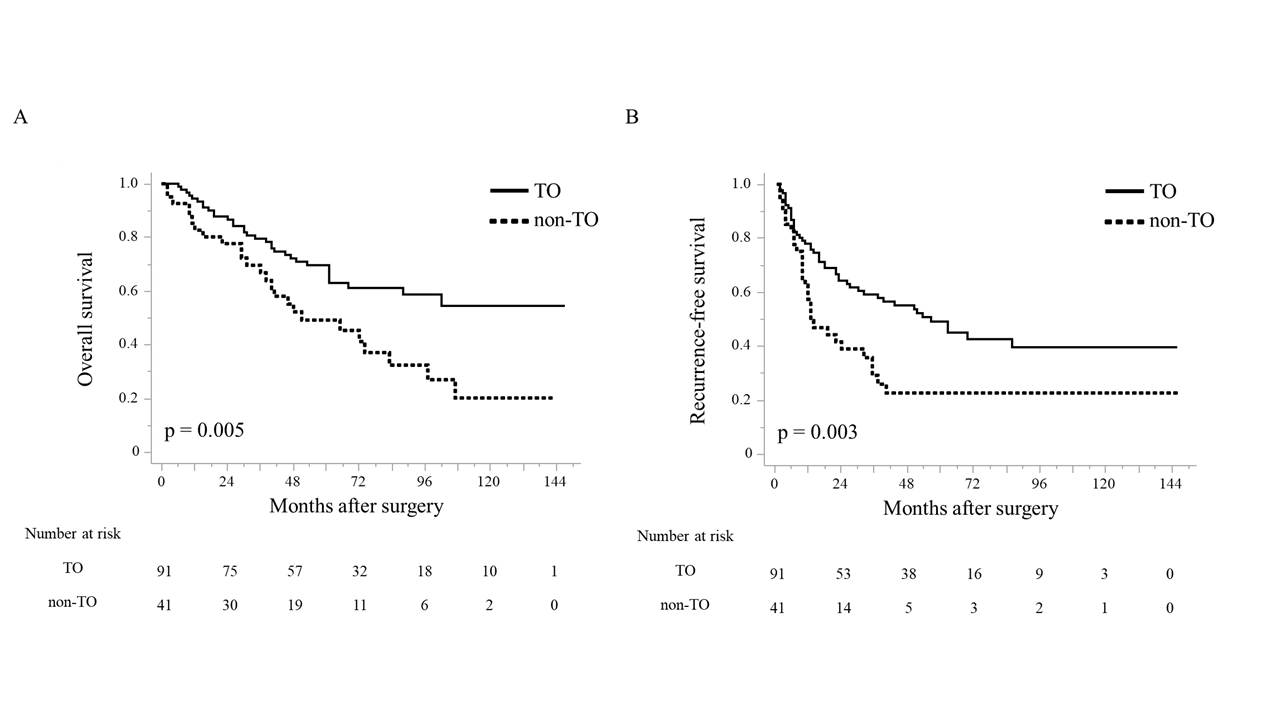

Supplement: Supplementary file 1 — Supplementary Material 1: Supplementary Fig. 1: Comparison of patient outcomes according to achievement of textbook outcome in the normal nutritional cohort. (a) Overall survival; (b) recurrence-free survival [file 423_2025_3703_MOESM1_ESM.tif]

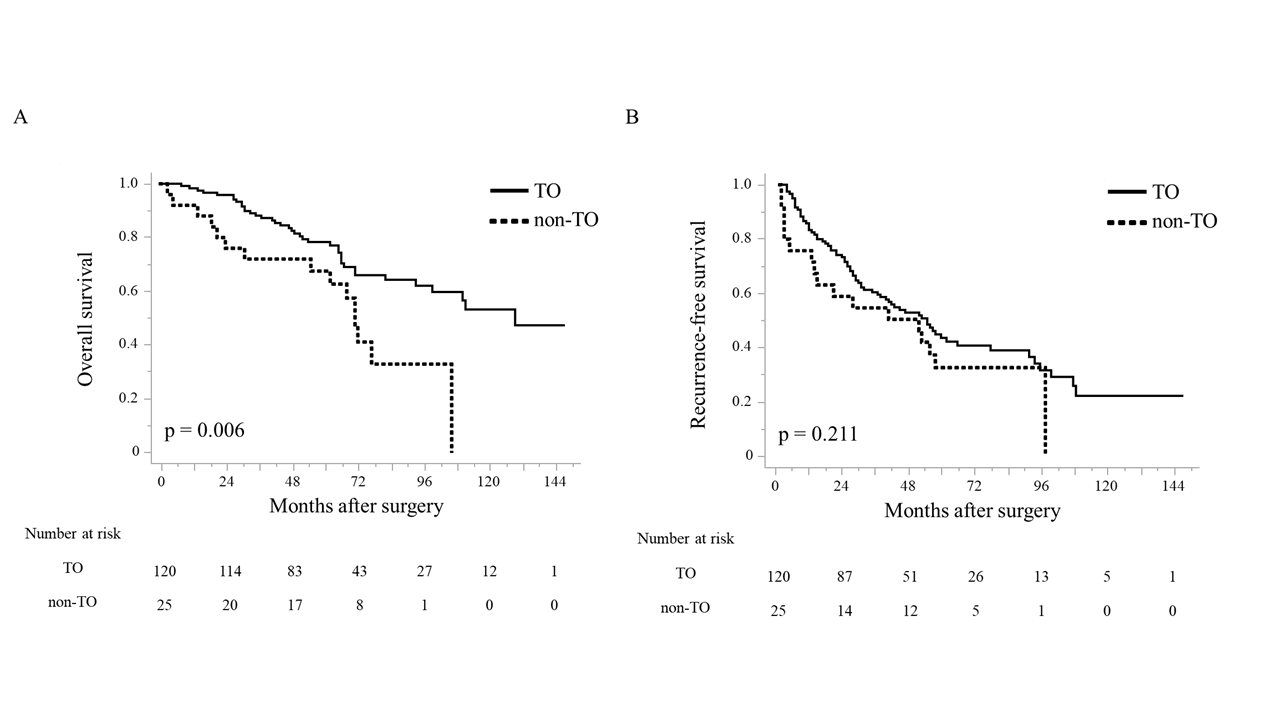

Supplement: Supplementary file 2 — Supplementary Material 2: Supplementary Fig. 2: Comparison of patient outcomes according to achievement of textbook outcome in the moderate malnutritional cohort. (a) Overall survival; (b) recurrence-free survival [file 423_2025_3703_MOESM2_ESM.tif]

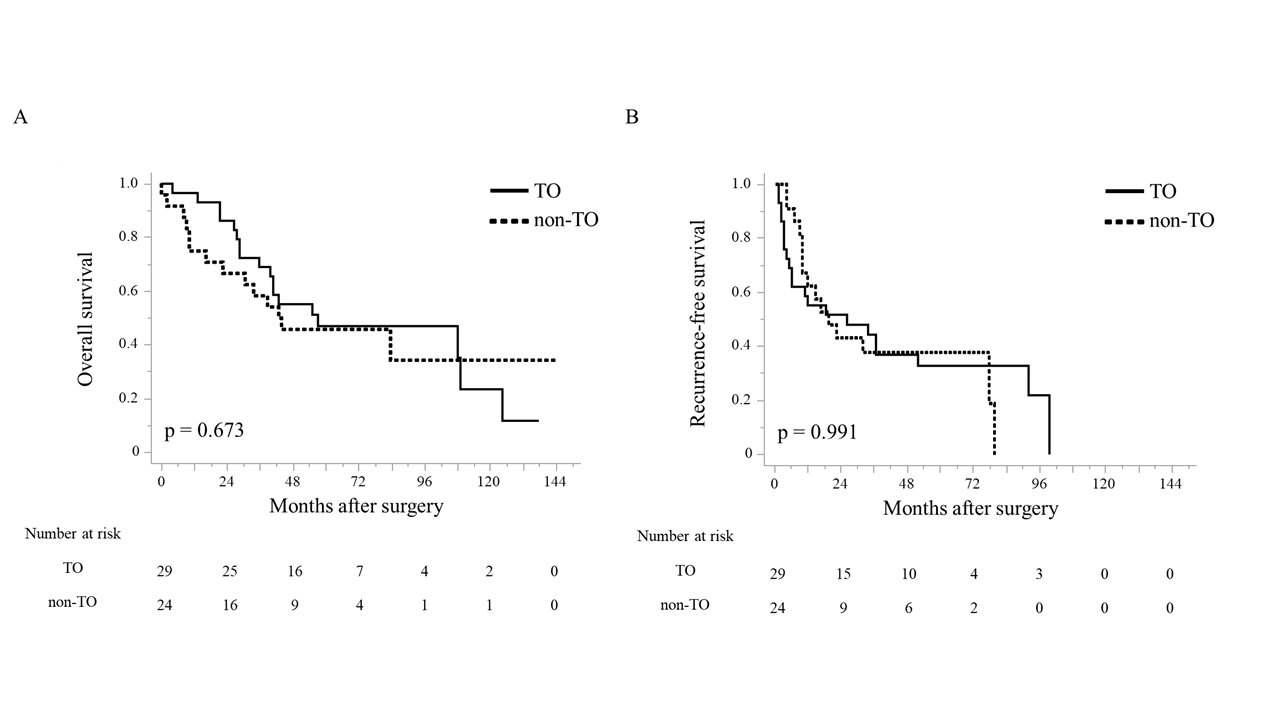

Supplement: Supplementary file 3 — Supplementary Material 3: Supplementary Fig. 3: Comparison of patient outcomes according to achievement of textbook outcome in the severe malnutritional cohort. (a) Overall survival; (b) recurrence-free survival [file 423_2025_3703_MOESM3_ESM.tif]
