# Supplementary material for: Achieving textbook outcome in liver resection for hepatocellular carcinoma: malnutrition’s pivotal role
Source: Langenbecks Arch Surg. 2025 Apr 23;410(1):139. doi: 10.1007/s00423-025-03703-x (PMC12018603; doi:10.1007/s00423-025-03703-x)
Supplement: Supplementary file 4 — Supplementary Material 4 [file 423_2025_3703_MOESM4_ESM.docx]

**LIST OF SUPPLEMENTARY INFORMATION**

**Supplementary Fig.1** Comparison of patient outcomes according to achievement of textbook outcome in the normal nutritional cohort

(a) Overall survival; (b) recurrence-free survival

**Supplementary Fig.2** Comparison of patient outcomes according to achievement of textbook outcome in the moderate malnutritional cohort

1. Overall survival; (b) recurrence-free survival

**Supplementary Fig.3** Comparison of patient outcomes according to achievement of textbook outcome in the severe malnutritional cohort

(a) Overall survival; (b) recurrence-free survival
